# Meetings of Societies

**Published:** 1940

**Authors:** 


### Meetings of Societies

### Bristol Medico-Chirurgical Society.

During the past Session three afternoon meetings have been held. On Wednesday, 13th December, Professor Samson Wright addressed the Society on the subject of "The Hypothalamus and Posterior Pituitary—Recent Clinical and Physiological Advances." On Wednesday, 10th January, Colonel Whitby spoke on "Chemotherapy—A survey of present knowledge concerning the sulphonamide group of drugs." On Thursday, 15th February, members of the Society visited the X-ray Therapy and Radium Department at the Bristol General Hospital, where Dr. F. Gower Bergin and his colleagues had arranged demonstrations of the various methods in use. That these "war-time" meetings have been welcomed is shown by the fact that so many attended.

